# The new platinum-based anticancer agent LA-12 induces retinol binding protein 4 *in vivo*

**DOI:** 10.1186/1477-5956-9-68

**Published:** 2011-10-31

**Authors:** Pavel Bouchal, Jiri Jarkovsky, Kristyna Hrazdilova, Monika Dvorakova, Iva Struharova, Lenka Hernychova, Jiri Damborsky, Petr Sova, Borivoj Vojtesek

**Affiliations:** 1 Masaryk University, Faculty of Science, Department of Biochemistry, Kotlarska 2, 611 37 Brno, Czech Republic; 2 Masaryk Memorial Cancer Institute, Regional Centre for Applied Molecular Oncology, Zluty kopec 7, 656 53 Brno, Czech Republic; 3 Masaryk University, Institute of Biostatistics and Analyses, Kamenice 3, 625 00 Brno, Czech Republic; 4 Masaryk Memorial Cancer Institute, Department of Oncological and Experimental Pathology, Zluty kopec 7, 656 53 Brno, Czech Republic; 5 University of Defence, Faculty of Military Health Sciences, Institute of Molecular Pathology, Trebesska 1575, 500 03 Hradec Kralove, Czech Republic; 6 Masaryk University, Faculty of Science, Department of Experimental Biology, Kamenice 5, 625 00 Brno, Czech Republic; 7 Centre of Biomolecular and Cellular Engineering, International Clinical Research Center, St. Anne's University Hospital Brno, Pekarska 53, 656 91 Brno, Czech Republic; 8 Platinum Pharmaceuticals a.s., Karasek 1, 621 33 Brno, Czech Republic

**Keywords:** (*OC*-6-43)-bis(acetato)(1-adamantylamine)amminedichloroplatinum (IV) (LA-12), plasma retinol-binding protein 4, RBP4, cisplatin, adamantylamine, proteomics

## Abstract

**Background:**

The initial pharmacokinetic study of a new anticancer agent (*OC*-6-43)-bis(acetato)(1-adamantylamine)amminedichloroplatinum (IV) (LA-12) was complemented by proteomic screening of rat plasma. The objective of the study was to identify new LA-12 target proteins that serve as markers of LA-12 treatment, response and therapy monitoring.

**Methods:**

Proteomic profiles were measured by surface-enhanced laser desorption-ionization time-of-flight mass spectrometry (SELDI-TOF MS) in 72 samples of rat plasma randomized according to LA-12 dose and time from administration. Correlation of 92 peak clusters with platinum concentration was evaluated using Spearman correlation analysis.

**Results:**

We identified Retinol-binding protein 4 (RBP4) whose level correlated with LA-12 level in treated rats. Similar results were observed in randomly selected patients involved in Phase I clinical trials.

**Conclusions:**

RBP4 induction is in agreement with known RBP4 regulation by amantadine and cisplatin. Since retinol metabolism is disrupted in many cancers and inversely associates with malignancy, these data identify a potential novel mechanism for the action of LA-12 and other similar anti-cancer drugs.

## Background

The platinum-based anti-cancer drug, cisplatin (cis-diamminedichloroplatinum(II)), is commonly used for treatment of various types of carcinomas, including breast, testicular, ovarian, head and neck or lung cancer [1], with significant anti-tumor activity. However, its clinical use is substantially complicated by numerous side effects such as nephrotoxicity, neurotoxicity and nausea as well as by intrinsic or acquired resistances. Significant efforts were therefore dedicated to develop novel platinum-based complexes to reduce the side effects of cisplatin, to overcome platinum resistance and to introduce novel mechanisms of anti-cancer action. Two derivatives, carboplatin (cis-diammine-(1,1-cyclobutanedicarboxylato)platinum(II)) and oxaliplatin (trans-[R, R-cyclohexane-1,2-diammine]oxalatoplatinum(II)), have been approved by the Food and Drug Administration for clinical use [2,3]. The requirement for their intravenous administration, in addition to substantial side effects, led to the development of a new generation of platinum-based drugs such as satraplatin ((*OC*-6-43)-bis(acetato)amminedichloro(cyclohexylamine)platinum(IV)), known also as JM216, the first orally administered platinum compound evaluated in clinical trials [2,4,5]. We studied the biological properties of an alternative platinum(IV) complex called LA-12, (*OC*-6-43)-bis(acetato)(1-adamantylamine)amminedichloroplatinum(IV), containing 1-adamantylamine instead of cyclohexylamine non-leaving ligand [6] which provides different chemical and biological properties and which has also entered into clinical evaluation. LA-12 has shown a higher cytotoxicity than satraplatin when tested on a panel of 14 cancer cell lines of various origins and different cisplatin sensitivities [6,7] and no cross-resistance with cisplatin [6,8]. LA-12 has also shown higher anti-tumor activity in comparison with cisplatin and satraplatin, favorable pharmacokinetics and relatively low acute toxicity in a panel of pre-clinical *in vivo *studies [9-11]. Generally, the cytotoxic mode of cisplatin action is mediated by its interaction with DNA to form DNA adducts, primarily intra-strand crosslink adducts, which activate several signal transduction pathways, including those involving ATR, p53, p73 and MAPK, and culminate in the induction of apoptosis [12]. The mechanisms of LA-12 action are not fully understood. There is evidence that exposure to LA-12 can disrupt cell proliferation and induce apoptosis more potently than cisplatin in both p53 dependent and independent manners [13,14]. In particular, LA-12 induces unique changes in the profile of gene expression compared to cisplatin, indicating a distinct mode of action resulting in the differential activation of both p53-dependent and p53-independent gene targets [15]. Most recently, LA-12 has been shown to have a greater inhibitory effect than cisplatin on heat shock protein 90 function [16].

To understand the molecular mechanisms of LA-12 action and identify serum markers for LA-12 activity in cancer patients, we performed dose-response and time-course studies using mass-spectrometry-based analysis to measure the proteomic profiles of rat plasma in response to LA-12 and compared them with the recent pharmacokinetic data [17]. Such an experimental design enables identification of LA-12 target proteins which could potentially serve as markers of LA-12 treatment, response and therapy monitoring. Using the surface-enhanced laser desorption-ionization time-of-flight mass spectrometry (SELDI-TOF MS) approach [18] we identified Retinol-binding protein 4 (RBP4) as significantly correlating with LA-12 level in both rat plasma and rat plasma ultrafiltrate and in the plasma of patients undergoing LA-12 treatment in Phase I clinical trials. In view of the known roles of retinol in controlling cellular differentiation and the abnormal expression of RBP4 in cancer [19], these data contribute to understanding LA-12 action as an anti-cancer agent and identify RBP4 as a serum marker for LA-12 activity.

## Methods

### Chemicals, animals and dosing

LA-12 was synthesized by Pliva-Lachema. 36 male albino Wistar-Hahn rats (6-8 weeks of age, 235-268 g) were kept under 12-h light/dark cycle with free access to water and standard diet for 13 days for acclimatization prior to experiments. All animal protocols were approved by the Institute's Animal Experimental Ethics Committee and animals were treated according to OECD guidelines. In the morning after overnight fasting, LA-12 was administered by a gastric gavage in a volume of 1 mL/kg of body weight as suspension in a 0.6% water solution of methylcellulose. Rats were randomly assigned to four LA-12 dosing groups of 9 rats dosed with 37.5 - 75 - 150 - and 300 mg LA-12/kg body weight each and, furthermore, to two of six blood sampling intervals (pre-dose and at 2, 8, 24, 48 and 72 h after dosage). Each combination of dosage and blood sampling time was thus represented by three animals. Two blood samples of 2 mL each were taken (at different times after dosage) from retro-orbital plexus under ether anesthesia. Blood was collected into polypropylene test tubes containing 0.75% K_3_EDTA (20 μL/mL of blood) and cooled in water bath (8°C) for 5 min. The samples were then centrifuged at 3000 *g *and 8°C for 10 min. The first aliquot (0.1 mL) of the supernatant was immediately frozen at -18°C while plasma ultrafiltrate was prepared from the remaining blood volume (see [17] for protocol) and stored at -18°C. After the second blood sampling interval, rats were killed by withdrawing whole blood from the abdominal artery.

### SELDI-TOF MS analysis

20 μL of rat plasma were denatured with 30 μL of sample solution (9 M urea, 2% 3-[(3-cholamidopropyl)dimethylammonio]-1-propanesulfonate (CHAPS)) for 30 min at room temperature (RT). Denatured plasma proteins were centrifuged (10,000 ×g/20 min/RT), the supernatant was mixed with 90 μL of IMAC binding buffer (Bio-Rad, USA) and loaded on IMAC-Cu SELDI chips according to manufacturer's instructions. After matrix (sinapinic acid) application, sample protein composition was analyzed using SELDI-TOF MS in PBS IIc Protein Chip Reader (Bio-Rad, USA) as described previously [18,20]. All samples characterized by one of four LA-12 doses and one of six time intervals, each obtained by three independent blood collections from individual animals (biological replicates) were measured twice; 4*6*3*2 = 144 averaged MS spectra were thus obtained. Peak clustering was performed with Biomarker Wizard software (Bio-Rad, USA) with signal/noise (S/N) > 5 and 5% minimum spectra detection in the first pass and then peaks with S/N > 3 in cluster mass window of range 0.3% were added; the valley depth was set to three fold of noise. Intensities of corresponding peaks across all six replicates (three biological replicates, each measured twice) were then averaged before quantitative statistical analysis.

### Platinum concentration measurement

Platinum concentration in both rat plasma and ultrafiltrate samples as well as in human plasma samples were determined by a validated method based on electrothermal atomic absorption analysis with Zeeman background correction, as described previously [17]. An AAnalyst 800 spectrometer (Perkin Elmer, Norwalk, CT, USA) with longitudinal AC-Zeeman-effect background correction with transversely heated graphite tube (THGA™) and autosampler AS 800 were used [17].

### Statistical analysis

The primary SELDI-TOF MS data consisting of six replicates (three biological replicates, each measured twice) were inspected for outliers and aggregated using average prior further statistical analyses. Values under detection limit were taken as half of this limit. Standard nonparametric descriptive statistics were adopted, i.e. median and percentile range.

The platinum concentrations in response to LA-12 dose and time since drug administration were statistically evaluated using Kruskal-Wallis test. The average intensities of all 92 protein peak clusters across the whole SELDI-TOF MS experiment were correlated with platinum concentration in corresponding samples using Spearman rank correlation coefficient. All analyses were performed using Statistica 9 software (StatSoft, Inc., USA).

### Protein identification

For identification of proteins that correlate with plasma platinum level, rat plasma samples containing highest levels of the proteins of interest were pre-separated using four IMAC Spin Columns (Bio-Rad, USA) (1 mg of total protein for each column) according to the manufacturer's instructions, with 2 × 100 μL of 250 mM imidazole in binding buffer as the elution buffer. Eluted protein mixtures were dialyzed overnight against 40 mM Tris-HCl buffer (pH 7.0), combined and dried under vacuum. The pellet of IMAC pre-separated proteins was resolubilized in 80 μL of sample solution containing 10% acetonitrile and 0.1% trifluoroacetic acid for further reverse phase-liquid chromatography fractionation. This fractionation was performed on an Agilent HP 1100 HPLC system (Agilent Technologies, Santa Clara, CA, USA) using a Discovery Bio Wide Pore C18 column (10 cm × 2.1 mm, 5 μm particle size; Sigma-Aldrich Corp., St. Louis, MO, USA) with a 2 cm guard precolumn. Separations were performed at 35°C, mobile phase A consisted of 0.1% trifluoroacetic acid in water while mobile phase B consisted of 0.1% trifluoroacetic acid in acetonitrile. The proteins were eluted using a linear gradient of mobile phase B (0% to 91% B in 15 min, then 91% B to 96% B in 18 min, then 96% B to 100% B in 2 min) followed by elution using 100% mobile phase B in 10 min; the flow rate was 100 μL/min (following the method of Moshkovskii *et al*. [21], with several modifications). Sixty collected fractions (60 μL each) were dried under vacuum and resolubilized in 20 μL 10% acetonitrile and 0.1% trifluoroacetic acid for protein profile determination on SELDI-TOF MS (2 μL of each resolubilized fraction was analyzed on NP-20 chips). The proteins were redissolved in 20 μL of sample buffer (consisting of 12% SDS, 6% mercaptoethanol, 30% glycerol, 0.05% Coomassie Brilliant Blue G-250 and 150 mM Tris-HCl, pH 7.0), heated (95°C/3 min), centrifuged (16,000 × g/20 min/4°C) and separated using tricine SDS-PAGE on PROTEAN II XL apparatus (Bio-Rad, USA) according to Schägger [22]. The gel - consisting of 4% sample loading gel, 10% spacer gel and 16% separation gel - was stained using colloidal Commassie Blue [23]. The bands with appropriate molecular weight were cut out and digested by trypsin as described previously [24]. Mass spectra were recorded in positive reflectron mode on a 4800 MALDI TOF/TOF™ mass spectrometer (Applied Biosystems, Framingham, MA, USA) equipped with an Nd:YAG laser (335 nm) using 3-7 ns pulse and with 200-Hz firing rate. Delayed extraction was used in all experiments being optimized for m/z = 2100 in MS mode. The maximum pulse energy was 23 μJ, it was attenuated appropriately for sample analysis. Accelerating voltage of the ion source was set to 20 kV in the MS mode. In the MS/MS mode, the accelerating voltage was 8 kV; it was modified after ion selection so the ions passing the collision cell possessed 1 keV of kinetic energy; the accelerating voltage rose to 15 kV after ions passed the collision cell. Dual microchannel plate detector voltage was set to 1.86 kV in MS mode and 2.10 kV in MS/MS mode. MS spectra were acquired in the mass range of 800-4000 m/z and calibrated internally using the monoisotopic [M+H]^+ ^ions of trypsin autoproteolytic fragments (842.509 Da and 2211.104 Da). Peak detection was performed using the internal algorithm of the 4000 Series Explorer™ Software (version 3.6; Applied Biosystems) with signal-to-noise ratio (S/N) higher than 55 in MS mode and S/N > 20 in MS/MS mode using cluster area optimization feature. Up to 5 precursors from MS spectra with S/N > 100 were automatically selected for MS/MS fragmentation analysis using the interpretation method of the Explorer™ Software. MS/MS acquisition of precursors was controlled according to decreasing S/N value. The isolation parameter for precursor selection was set to 200 as for the resolution of ion gating mechanism.

The mgf peak lists were generated from mass spectra using the Peaks-to-Mascot function incorporated in the Explorer™ software. From MS analysis, peaks in the m/z range of 800-4000 and with S/N > 30 were included in the mgf peak list. From MS/MS analysis, peaks fulfilling two following criteria were included in the mgf peak lists: (i) S/N > 15 and (ii) the m/z range between 68 and the value of 50 m/z units lower than precursor's m/z value. The peak lists containing both MS and MS/MS data were submitted through Mascot Daemon (ver. 2.1.0) to Mascot Server (local installation of database search engine, ver. 2.1.04). Parameters for combined search (MS and MS/MS data) were as follows: database - UniProt SwissProt (release 2011_01); taxonomy - all entries; enzyme - trypsin; allowed missed cleavages - 1; fixed modifications - carbamidomethyl (C); variable modifications - oxidation (M), pyro-cmC (N-term camC), pyro-glu (N-term E), pyro-glu (N- term Q); peptide tolerance - 30 ppm; MS/MS tolerance - 250 mmu; peptide charge - (+1); monoisotopic masses; instrument - MALDI-TOF-PSD; no restrictions on protein molecular weight and pI value were applied. The hits passing the following criteria were concluded as a successful protein identification: (i) protein score C.I. = 100%, (ii) total ion score C.I. = 100%, and (iii) and at least two successfully fragmented peptides with ion score C.I = 100% each.

### Determination of RBP4 level in plasma of patients from LA-12 clinical evaluation

Patients with solid tumors undergoing Phase I clinical trials were administered orally by LA-12. Blood was collected into test-tubes containing 40 μL of 3.75% K_3_EDTA before administration and 0.5-1-2-4-8 h after administration, centrifuged at 4000 rpm at 4°C for 10 min. The plasma was transferred into labeled polypropylene test-tubes and immediately frozen and stored in a freezer (-18°C). Plasma samples from patients containing 30 μg of total protein were separated by SDS-PAGE and transferred onto nitrocellulose membranes which were incubated with primary antibody (Anti-RBP4, Sigma-Aldrich, USA, cat. No. HPA001641), detected with peroxidase conjugated anti-rabbit IgG (Dako, UK) and ECL detection reagents (GE Healthcare, Sweden). Band intensities were quantified using QuantityOne 4.6.1 software (Bio-Rad, USA).

## Results and discussion

**Figure 1:**
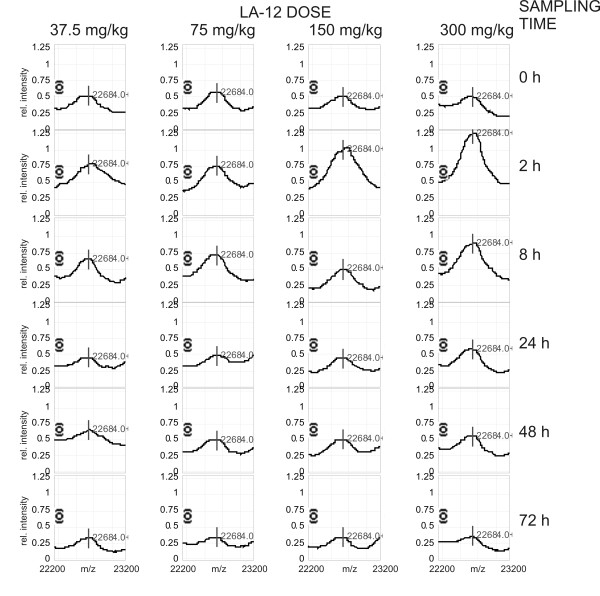
**Representative overview of peak 75 intensity (m/z = 22684, identified as rat RBP4 protein) in response to LA-12 dose and time left after dosage (= sampling time)**. Peaks presented in each column correspond to the same LA-12 dose while peaks in a similar row correspond to the same sampling time. The peak elevation with a maximum two hours after LA-12 dosage, responsively to LA-12 dose and in overall correlation with Pt level, is a key observation in this study.

**Figure 2:**
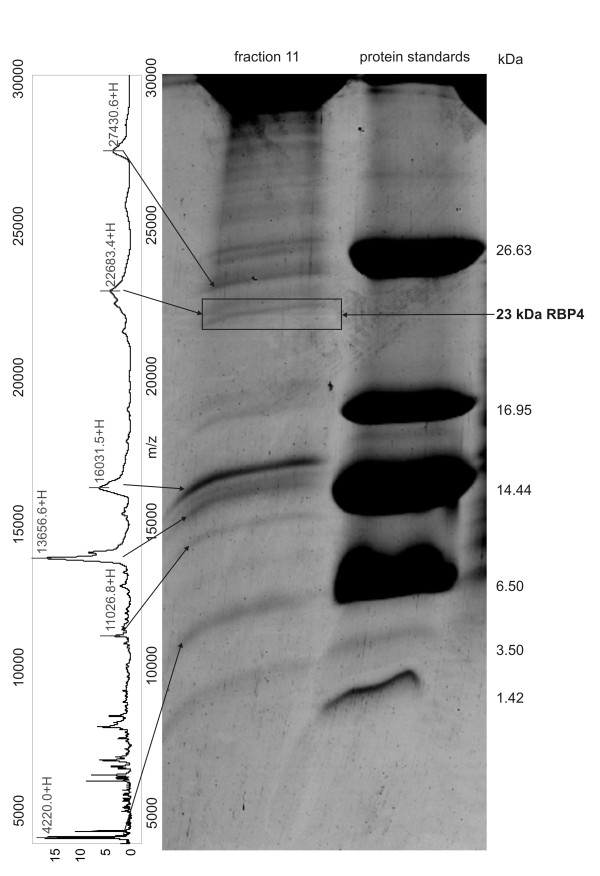
**The process of identification of SELDI-TOF MS peak No. 75**. After two-dimensional protein separation, the RP-LC fraction No. 11 containing protein corresponding with peak No. 75 was separated on tricine SDS-PAGE. The corresponding gel band was identified by comparison of molecular masses as well as by comparison of SELDI-TOF MS spectrum with the electrophoregram. The band was then excised from the gel, analyzed using mass spectrometry and identified as RBP4 protein (UniProt accession No. P04916).

**Figure 3:**
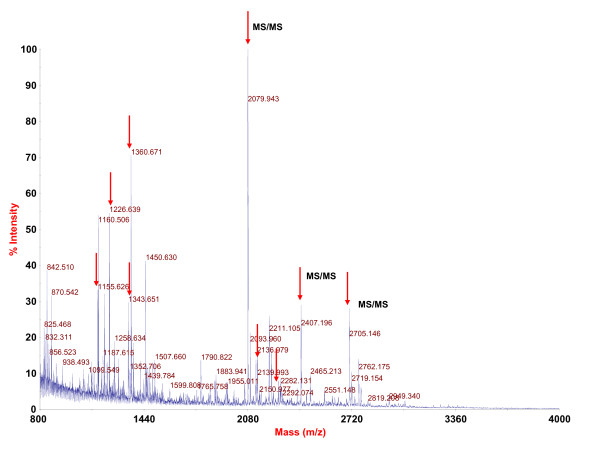
**MS spectrum of rat RBP4 protein (Plasma retinol-binding protein precursor (P04916, RET4_RAT)**. MALDI MS spectrum of tryptic peptides extracted from excised and digested protein gel band (Figure 2) was measured in positive reflectron mode. Arrows indicate the peptides identified in PMF analysis. Three peaks marked with "MS/MS" were successfully fragmented and identified with significant score (see Additional file 5 for MS/MS peptide spectra).

Platinum concentrations (corresponding to LA-12 level) were measured in plasma of rats administered four different doses of LA-12 (37.5 - 75 - 150 - and 300 mg LA-12/kg body weight). The measurements were conducted for the samples collected at 0 - 2 - 8 - 24 - 48 - 72 h after LA-12 dosage. As each sample was available in three biological replicates (4 dosages × 6 times × 3 rats), 72 measurements of platinum concentration in rat plasma were performed. Platinum concentration reached its maximum 2 h after administration and then started to decrease (see Additional file 1 for the statistically evaluated results). In addition, ultrafiltrates of the plasma were prepared from the samples collected at 0 - 2 - 8 h after LA-12 dosage and Pt concentrations were measured with maximum at 2 h (Additional file 1). The results serve as a complementary set of Pt concentrations reflecting a free, non-protein platinum content. In parallel, SELDI-TOF MS proteomic profiles of the 72 plasma samples were measured (each in two analytical replicates), 144 MS spectra were thus obtained. Additional file 2 shows a typical SELDI-TOF MS result. In total, 92 protein/peptides were detected and quantified using Biomarker Wizard software in each MS spectrum across the experiment - see Additional file 3 for details of these 92 protein and peptide clusters. Using Spearman correlation statistical analysis, we identified the protein/peptide peaks with intensities that correlated with LA-12 dose and time between the dosage and blood sampling. The values of Spearman correlation for all detected peaks are presented in Additional file 3. Only protein peak No. 75 (m/z = 22684) exhibited a statistically significant positive correlation with platinum level in both rat plasma (Spearman correlation coefficient 0.248) and plasma ultrafiltrate (Spearman correlation coefficient 0.342). See Figure 1 for its intensity in response to LA-12 dose and sampling time. The experiment confirming that quantification based on this peak was within the linear range of quantification is presented in Additional file 4. To reveal the sequence identity of this protein peak, we used a three-dimensional separation method followed by tandem mass spectrometry (MALDI-MS/MS). In principle, plasma proteins were first pre-separated on IMAC Spin columns to obtain the same SELDI-TOF MS protein composition as measured using analytical IMAC 30 chips. Second, sample complexity was reduced by liquid chromatography with reverse phase column (RP-LC). Individual RP-LC fractions were then analyzed using SELDI-TOF MS (normal phase NP-20 chip surface) to identify the fractions containing proteins of interest. Only one fraction (No. 11) contained the protein peak with m/z = 22684 (see Additional file 5 for confirmatory SELDI-TOF MS spectra of all fractions in this m/z region). Third, fraction No. 11 was then separated by tricine SDS-PAGE. The corresponding protein band in the gel was identified using protein profile matching and by comparison of intact protein molecular weights as shown in Figure 2. Sequence identity of this LA-12 positively correlating protein was then obtained using MALDI-MS/MS as Retinol-binding protein 4 (RBP4, UniProt accession No. P04916, Mascot score 472, E-value 3.3 × 10^-42^, sequence coverage 55%, protein identification based on 9 tryptic peptides and three of them were confirmed by MS/MS analysis). See Figure 3 for corresponding for MALDI-MS spectrum, Table 1 for overview of all identified peptides and Additional file 6 for MS/MS spectra of identified peptides.

**Table 1:** Rat RBP4 protein identification data

Plasma retinol-binding protein precursor (RBP4)									
UniProt Accession: P04916 (RET4_RAT)									
Protein Score		472							
Protein Score C.I. %		100							
Total Ion Score		394							
Total Ion Score C.I. %		100							
Sequence Coverage %		55							
E-value		3.3 × 10^-42^							
									
Obsrv. Mass	Calc. Mass	± Da	Start Seq.	End Seq.	Miss Cleav.	Ion Score	C.I. %	E-value	Peptide Sequence+Modification
1155.6251	1154.6124	0.0054	38	47	0	---	---	---	R.FSGLWYAIAK.K
1226.6394	1225.6243	0.0078	108	117	0	---	---	---	K.YWGVASFLQR.G
1343.6510	1342.6299	0.0138	172	181	1	---	---	---	R.QRQEELCLER.Q + Pyro-glu (N-term Q)
1360.6714	1359.6565	0.0077	172	181	1	27	39	0.85	R.QRQEELCLER.Q
2079.9431	2078.9367	-0.0009	140	157	0	148	100	2.9e-13	R.LQNLDGTCADSYSFVFSR.D
2129.0942	2128.1138	-0.0269	1	20	0	---	---	---	-.MEWVWALVLLAALGGGSAER.D
2279.1006	2278.1004	-0.0071	49	68	0	---	---	---	K.DPEGLFLQDNIIAEFSVDEK.G
2407.1960	2406.1954	-0.0067	48	68	1	132	100	2.1e-11	K.KDPEGLFLQDNIIAEFSVDEK.G
2705.1455	2704.1500	-0.0118	118	139	0	87	100	8.6e-8	R.GNDDHWIIDTDYDTFALQYSCR.L

Table includes protein name, UniProt accession number and entry name, Protein Score, Protein Score C.I. %, Total Ion Score, Total Ion Score C.I. %, Sequence Coverage %, E-value; list of peptides matched to the identified protein with their observed and calculated m/z values, error of the measurement (Da), start and end position in protein sequence, number of missed cleavages, Ion Score, C.I. % for peptides confirmed by MS/MS, their E-value, sequences of identified peptides and note about their modification. The data correspond to gel piece excised from the gel as shown in Figure 2, no other protein was identified with significant score in tryptic digest of this gel band.

RBP4 is a plasma protein characterized by Kanai and Goodman [25] as a transport protein for retinol produced in hepatocytes to extrahepatic tissues. Retinoids (retinol, all-trans retinoic acid and related signaling molecules) are involved in cellular differentiation pathways and induce differentiation of various types of stem cells [19]. Components of the retinol pathway are disrupted in cancer, which suggests that a reduction of retinoid signaling is involved in tumor development. This applies to RBP4 and RBP1, amongst others, which are down-regulated by methylation in cancer [26]. Also, RBP4 is known to influence the differentiation of adipocytes [27] and to mediate insulin resistance through its functional relationship to glucose transporter 4 [28].

In principle, the RBP4 correlation with LA-12 observed in our data might be caused by two major reasons: (i) by formation of a RBP4/LA-12 complex analogous to that of RBP4/retinol, or (ii) by induction of RBP4 protein expression. In order to explore the first hypothesis, we performed a molecular modeling of the RBP4/LA-12 complex to investigate whether LA-12 is able to bind non-covalently into the retinol binding site in the RBP4 structure. In such a case, RBP4 would hypothetically serve as a LA-12 transport protein analogously to the retinol. The modeling revealed that the central cavity of the protein is too small for binding of LA-12 and the protein would have to undertake a significant conformational change to form a cavity capable of binding LA-12. The comparative analysis of the apo and holo structures of RBP4 [29] revealed some flexibility in the loop composed of residues 34-37 (Additional file 7). However, the other side of the binding cavity is made of a rigid β-barrel, making significant enlargement of the cavity unlikely. The above observations are supported by the fact that no peak with 553 Da mass shift (corresponding to LA-12 molecular weight) was detected in the surroundings of peak No. 75 in SELDI-TOF MS spectra (Additional file 2). It is thus unlikely that a hypothetical RBP4/LA-12 complex plays a significant role in LA-12 and/or RBP4 function.

On the other hand, the second hypothesis of direct or indirect induction of RBP4 expression by LA-12 is supported by induction of RBP4 by amantadine, a drug bearing an adamantine group closely related to that present in LA-12 [30]. Additional indirect evidence was indicated by Hung *et al*. who reported increased level of urinary RBP4 in mice after cisplatin treatment [31]. This suggests similar mechanisms for RBP4 induction by both LA-12 and cisplatin. The time dependence of the RBP4 level in rat plasma also corresponds well with the protein stability which has been estimated for approximately 30 h [32].

**Figure 4:**
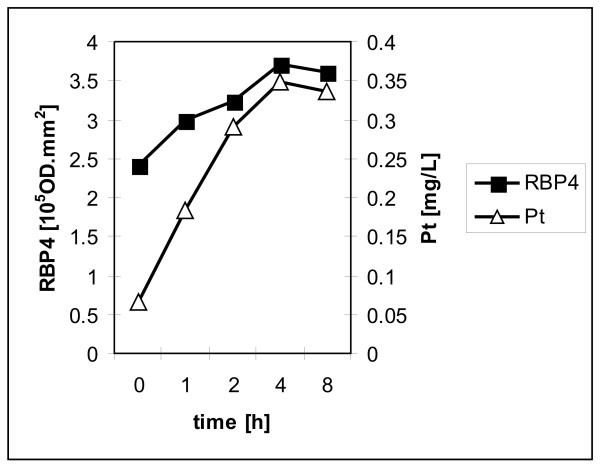
**Similar trends in average platinum concentration and RBP4 level (measured as average integral optical density by western blotting with immunochemical detection) in response to sampling time in patients'plasma**. Data from individual patients are presented in Additional file 8.

To independently verify that the identified peak in the rat experimental system is indeed RBP4 and to investigate RBP4 induction by LA-12 in humans, we analyzed plasma samples using western blotting with an RBP4 specific antibody. Although this antibody to human RBP4 was not effective in rat samples, we demonstrated that circulating RBP4 levels correlated well with platinum levels in human plasma of 12 randomly selected patients involved in Phase I clinical trials of LA-12 (see Figure 4 and Additional file 8).

Functionally, RBP4 is aberrantly expressed in a number of human cancers, with promoter hypermethylation leading to down-regulation of expression in esophageal squamous cancer [26]. Most recently, Lorkova *et al*. [33] reported a decreased level of RBP4 in sera of ovarian cancer patients using two-dimensional gel electrophoresis and confirmed this observation *via *western blotting and ELISA. In view of the roles of retinol in cellular differentiation, the lack of RBP4 expression is thought to be involved in maintaining the undifferentiated nature of cancer cells [19]. Thus, in addition to acting as a marker, elevated RBP4 levels following treatment with LA-12 may have functional importance through the restoration of retinol-induced signaling pathways in cancer cells. As such, these data open very interesting, much more complex and not yet investigated hypothesis on RBP4 involvement in cancer development and the mechanisms of action of LA-12 and similar anti-cancer drugs.

## Conclusions

Using mass spectrometry-based proteomics, we identified induction of RBP4 by the new anticancer agent LA-12 in both rat and human plasma. RBP4 is a plasma protein involved in the transport of retinol, which serves as a differentiation-inducing molecule in various stem cells and is aberrantly expressed in cancer.

## Competing interests

Petr Sova is a co-author of patents in the field of LA-12.

## Authors' contributions

PB supervised the SELDI-TOF MS study, developed the protocol for protein identification and prepared the manuscript for publication. JJ performed the statistical data analysis. KH prepared the plasma samples and measured them using SELDI-TOF MS. MM has carried out the protein separations before MALDI-MS/MS identification. IS validated the RBP4 expression using western blotting. LH identified the RBP4 protein via MALDI-MS/MS. JD carried out the modelling of LA-12/RBP4 interactions. PS have made substantial contributions to study design. BV contributed to manuscript preparation revising it critically for important intellectual content and have given final approval of the version to be published. All authors read and approved the final version of the manuscript.

## Authors' information

The authors' team is based at Masaryk Memorial Cancer Institute, Brno, Czech Republic and at cooperating institutes (Masaryk University, University of Defence and Platinum Pharmaceuticals). The interdisciplinary team consists of specialists in cancer proteomics (PB-proteomics group leader; IS, KH, MM), biostatistics (JJ), proteomics mass spectrometry (LH), molecular modeling and protein engineering (JD), tumor biology and immunochemistry (BV) as well as of LA-12 project leader (PS). BV is a scientific director of Regional Centre of Applied Molecular Oncology (RECAMO, http://www.recamo.cz).

## Supplementary Material

Additional file 1 **Concentration profiles of platinum in rat plasma and plasma ultrafiltrate in response to LA-12 dose and to time after LA-12 dosage (Kruskal-Wallis ANOVA)**. The platinum concentrations (corresponding to LA-12 level) measured in the plasma samples of the rats dosed with four different LA-12 doses Click here for file

Additional file 2 **The typical SELDI-TOF MS spectrum of plasma from rats dosed with LA-12**. The spectra in m/z range 2000-80000 (A), 2000-30000 (B) and 20500-25600 (C) are shown. The arrows indicate the position of peak cluster No. 75 (m/z = 22684) identified as RBP4 protein. Click here for file

Additional file 3 **Overview of peptide and protein clusters and their correlation coefficients with platinum level in rat plasma and plasma ultrafiltrate**. Overview of peptide and protein clusters and their correlation coefficients with platinum level in rat plasma and plasma ultrafiltrate. Click here for file

Additional file 4 **Quantification of m/z = 22684 peak in primary proteomic study lies within linear range of quantification**. An independent experiment using beta-lactoglobulin A protein standard was performed to confirm that RBP4 quantification using SELDI-TOF MS lies within linear range of quantification. Click here for file

Additional file 5 **Overview of protein fractions obtained by reverse-phase protein fractionation as measured by SELDI-TOF MS (NP-20 surface)**. Overview of protein fractions obtained by reverse-phase protein fractionation as measured by SELDI-TOF MS (NP-20 surface). Protein with m/z = 22684 was detected in fraction 11 only and confirmed in a mixture of fractions 11,12,13 and 14. Click here for file

Additional file 6 **MS/MS spectra of rat RBP4 identified peptides**. Figure A. MS/MS spectrum of peptide m/z = 2079.943. Peptide was selected from MS spectrum of Plasma retinol-binding protein Precursor P04916 (see Figure 3 in the main text). Measured fragment series y and b are marked in spectrum and their m/z values are listed in table together with amino acid sequence of peptide. Asterisk indicates carbamidomethylation of cysteine residue. Figure B. MS/MS spectrum of peptide m/z = 2407.196. Peptide was selected from MS spectrum of Plasma retinol-binding protein precursor P04916 (see Figure 3 in the main text). Measured fragment series y and b are marked in spectrum and their m/z values are listed in table together with amino acid sequence of peptide. Figure C. MS/MS spectrum of peptide m/z = 2705.146. Peptide was selected from MS spectrum of Plasma retinol-binding protein precursor P04916 (see Figure 3 in the main text). Measured fragment series y and b are marked in spectrum and their m/z values are listed in table together with amino acid sequence of peptide. Asterisk indicates carbamidomethylation of cysteine residue. Click here for file

Additional file 7 **The structural alignment of the ribbon models of the apo structure of human plasma retinol-binding protein (PDB ID **1BRT**) and its holo structure (PDB ID **1BRP**) in the complex with the native ligand retinol**. The apo structure is shown in magenta, the holo structure is in blue and the retinol is colored by atom type. The most significant difference between two forms of the protein is due to the conformational change involving residues from 34 to 37 (black arrow). The figure was prepared using the software PyMol v0.99 (DeLano Scientific LCC, South San Francisco, CA, USA). Click here for file

Additional file 8 **The correlation between platinum and RBP4 protein levels in plasma from 12 patients undergoing the Phase I clinical trials**. Each patient is represented by one chart (A-L). Left axis and triangles corresponds with platinum level while right axis and squares stand for RBP4 level. Original western blotting data are presented above each chart. Click here for file
