# Supplementary material for: The new platinum-based anticancer agent LA-12 induces retinol binding protein 4 in vivo
Source: Proteome Sci. 2011 Oct 31;9:68. doi: 10.1186/1477-5956-9-68 (PMC3221626; doi:10.1186/1477-5956-9-68)

Additional file 1. Concentration profiles of platinum in rat plasma and plasma ultrafiltrate in response to LA-12 dose and to time after LA-12 dosage (Kruskal-Wallis ANOVA)

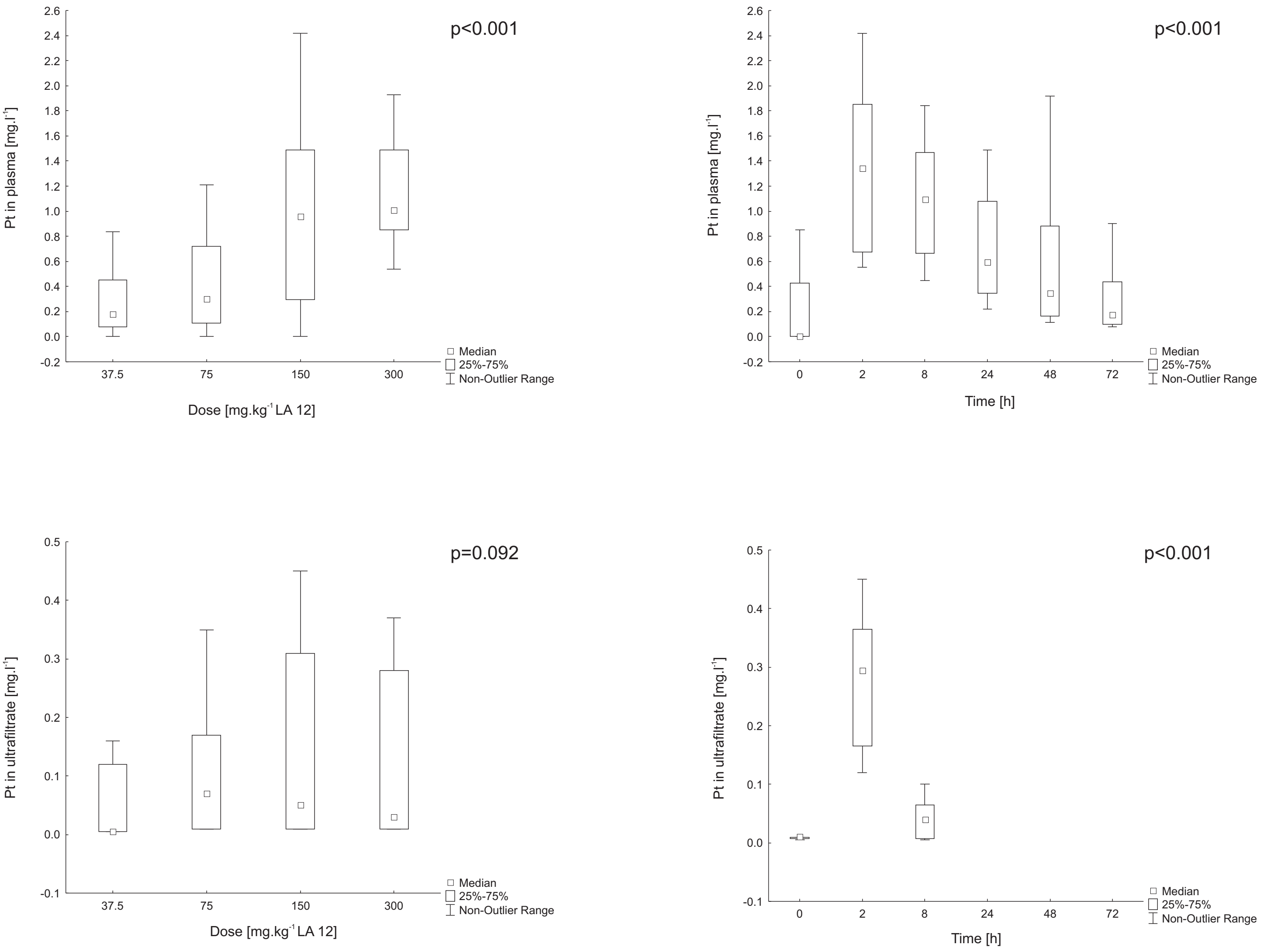

Supplement: Additional file 1 — Concentration profiles of platinum in rat plasma and plasma ultrafiltrate in response to LA-12 dose and to time after LA-12 dosage (Kruskal-Wallis ANOVA). The platinum concentrations (corresponding to LA-12 level) measured in the plasma samples of the rats dosed with four different LA-12 doses [file 1477-5956-9-68-S1.PDF]
