# Supplementary material for: The new platinum-based anticancer agent LA-12 induces retinol binding protein 4 in vivo
Source: Proteome Sci. 2011 Oct 31;9:68. doi: 10.1186/1477-5956-9-68 (PMC3221626; doi:10.1186/1477-5956-9-68)
Supplement: Additional file 2 — The typical SELDI-TOF MS spectrum of plasma from rats dosed with LA-12. The spectra in m/z range 2000-80000 (A), 2000-30000 (B) and 20500-25600 (C) are shown. The arrows indicate the position of peak cluster No. 75 (m/z = 22684) identified as RBP4 protein. [file 1477-5956-9-68-S2.PDF]

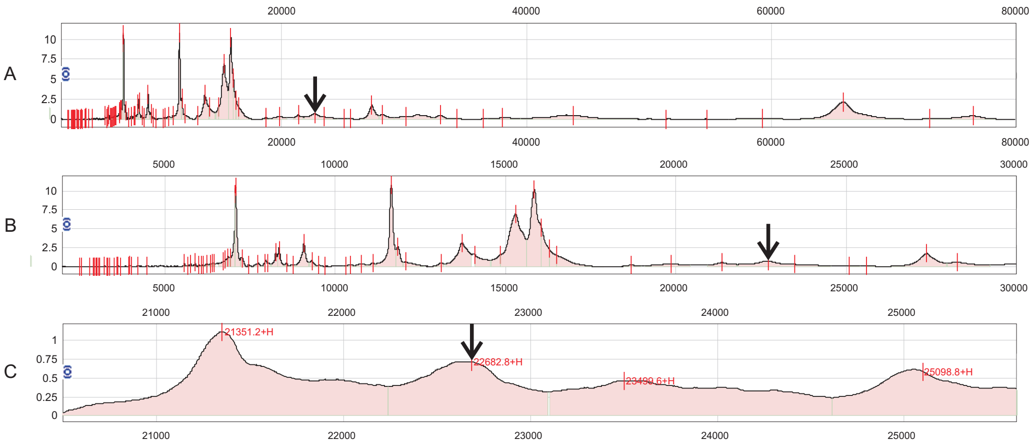

Additional file 2. The typical SELDI-TOF spectrum of plasma from rats dosed with LA-12. The spectra in m/z range 2000-80000 (A), 2000-30000 (B) and 20500-25600 (C) are shown. The arrows indicate the position of peak cluster No. 75 (m/z=22684) identified as RBP4 protein.
