# Supplementary material for: The new platinum-based anticancer agent LA-12 induces retinol binding protein 4 in vivo
Source: Proteome Sci. 2011 Oct 31;9:68. doi: 10.1186/1477-5956-9-68 (PMC3221626; doi:10.1186/1477-5956-9-68)
Supplement: Additional file 3 — Overview of peptide and protein clusters and their correlation coefficients with platinum level in rat plasma and plasma ultrafiltrate. Overview of peptide and protein clusters and their correlation coefficients with platinum level in rat plasma and plasma ultrafiltrate. [file 1477-5956-9-68-S3.PDF]

Additional file 3. Overview of peptide and protein clusters and their correlation coefficients with platinum level in rat plasma and plasma ultrafiltrate. Statistically significant correlations are marked in red. Peak 75 was identified as plasma retinol binding protein RBP4.

# of peptide/protein peak cluster	m/z	Spearman correlation coefficient for correlation with platinum level in rat plasma	Spearman correlation coefficient for correlation with platinum level in rat plasma ultrafiltrate
1	1061.5	0.036	-0.070
2	1099.2	0.029	-0.079
3	2513.2	0.017	0.031
4	2568.7	-0.061	-0.121
5	2795.0	0.010	0.045
6	2909.6	0.095	0.043
7	2926.4	0.056	0.090
8	2944.4	0.001	0.001
9	2994.7	0.147	0.316
10	3010.9	0.100	0.041
11	3020.6	0.115	0.000
12	3070.7	-0.038	-0.086
13	3091.6	0.211	0.098
14	3237.2	0.166	0.191
15	3302.5	0.194	0.065
16	3325.5	-0.061	-0.138
17	3385.8	-0.001	-0.063
18	3523.2	-0.011	-0.169
19	3605.2	-0.004	-0.032
20	3625.7	-0.038	0.163
21	3713.8	0.080	0.219
22	3954.6	-0.120	-0.213
23	3971.8	0.121	-0.058
24	4014.5	0.159	0.020
25	4209.5	0.186	-0.038
26	4489.5	0.113	**0.389**
27	5583.7	0.041	-0.195
28	5679.4	0.019	0.168
29	5771.3	0.066	-0.273
30	5893.1	0.159	0.022
31	6016.5	0.214	0.118
32	6075.4	0.134	-0.062
33	6130.8	0.148	0.254
34	6244.4	0.138	0.034
35	6334.9	0.053	-0.098
36	6402.8	0.057	-0.066
37	6441.7	0.070	0.126
38	6724.0	0.088	0.110
39	6785.0	0.042	0.060
40	6848.3	-0.164	-0.315
41	6920.4	0.057	**0.349**
42	7073.5	0.219	**0.506**
43	7085.8	0.215	0.165
44	7282.3	0.066	-0.321
45	7470.3	0.120	**-0.352**
46	7743.4	0.227	-0.136
47	7936.2	0.184	-0.246
48	8038.8	0.149	**-0.329**
49	8265.3	0.132	-0.171
50	8366.4	0.206	-0.245
51	8588.0	0.199	-0.251
52	8774.2	0.050	-0.268
53	9089.0	-0.091	**-0.546**
54	9330.0	0.197	-0.003
55	9515.9	0.064	-0.007
56	9718.5	0.053	0.035
57	10316.2	-0.034	0.072
58	10452.0	-0.131	0.215
59	10768.3	-0.094	**0.405**
60	11100.8	-0.090	**0.414**
61	11652.1	0.225	0.177
62	11851.6	0.149	0.239
63	12066.4	0.020	0.265
64	13128.3	-0.170	0.016
65	13720.8	0.130	0.127
66	14091.4	-0.225	**0.402**
67	14843.3	-0.185	0.164
68	15300.4	**-0.342**	-0.144
69	15855.4	**-0.232**	**-0.333**
70	16061.6	**-0.336**	-0.263
71	16285.5	**-0.333**	-0.138
72	16504.7	**-0.254**	-0.226
73	18687.3	-0.118	0.275
74	21354.2	0.000	-0.084
75	22684.8	**0.248**	**0.342**
76	23501.0	**0.297**	-0.148
77	25100.5	**0.306**	-0.209
78	25608.1	**0.310**	0.138
79	27363.5	0.153	0.191
80	28259.8	0.029	-0.041
81	30166.5	0.155	0.159
82	32967.6	0.123	-0.133
83	34293.9	-0.128	0.325
84	36535.1	-0.151	0.243
85	38028.5	**0.350**	0.230
86	43834.3	0.050	0.018
87	51407.7	-0.084	0.309
88	54749.5	0.110	0.161
89	59310.2	0.098	-0.130
90	65823.1	0.155	-0.173
91	72927.8	-0.206	0.107
92	76444.2	0.216	-0.029
