# Supplementary material for: The new platinum-based anticancer agent LA-12 induces retinol binding protein 4 in vivo
Source: Proteome Sci. 2011 Oct 31;9:68. doi: 10.1186/1477-5956-9-68 (PMC3221626; doi:10.1186/1477-5956-9-68)
Supplement: Additional file 4 — Quantification of m/z = 22684 peak in primary proteomic study lies within linear range of quantification. An independent experiment using beta-lactoglobulin A protein standard was performed to confirm that RBP4 quantification using SELDI-TOF MS lies within linear range of quantification. [file 1477-5956-9-68-S4.PDF]

QUANTIFICATION OF m/z=22684 PEAK IN PRIMARY PROTEOMIC STUDY LIES WITHIN THE LINEAR RANGE OF QUANTIFICATION

Aim

We have performed quantitative measurements on the series-of-dilutions of beta-lactoglobulin A using SELDI-TOF MS to confirm that RBP4 quantitative masurements performed within the primary proteomic study using SELDI-TOF MS lie in the linear range of quantification. A mass of the protein chosen (beta-lactoglobulin A bovine, m/z=18363.3) is the closest mass to those of RBP4 mass (m/z=22684) among the standards available from the instrument manufacturer.

Material and methods

Content of one tube of lyophylized beta-lactoglobulin A protein standard (Ciphergen/Bio-Rad product No. 4003-1014) was dissolved in 200 µl of distilled water (standard stock solution). Based on initial measurements of repeatedly 10-fold diluted protein series (data not shown), we found that 0.5-4 µl of the standard stock solution provide the peak intensities in a range corresponding to those of RBP4 as measured in the primary proteomic experiment (Fig. 1 in main text of the manuscript). For the confirmatory experiment, the amounts of 4 µl, 2 µl, 1 µl and 0.5 µl of the standard stock solution were denatured with 30 µL of 9 M urea + 2% CHAPS and diluted with 90 µL of IMAC binding buffer (Bio-Rad, USA). From this point, the analysis on IMAC30 chip surface was performed the same way as for the plasma samples in the initial proteomic experiment (see Material and methods section in the main text of the article). All measurements were performed in hexaplicates (each replicate measured on different chip) in agreement with the design of primary proteomic study.

Results

The complete series of SELDI-TOF MS spectra within the confirmatory experiment is presented in Supplementary Figure 1.
Supplementary Figure 2 shows that peak intensity depends linearly with R^2=0.9846 on the protein concentration and for m/z=18363.3 of beta-lactoglobulin A which is close to those of RBP4.

Conclusion

The data support the reliablity and accuracy of quantitation for RBP4 in the concentration range detected in rat plasma and for the experiment type presented in this communication.

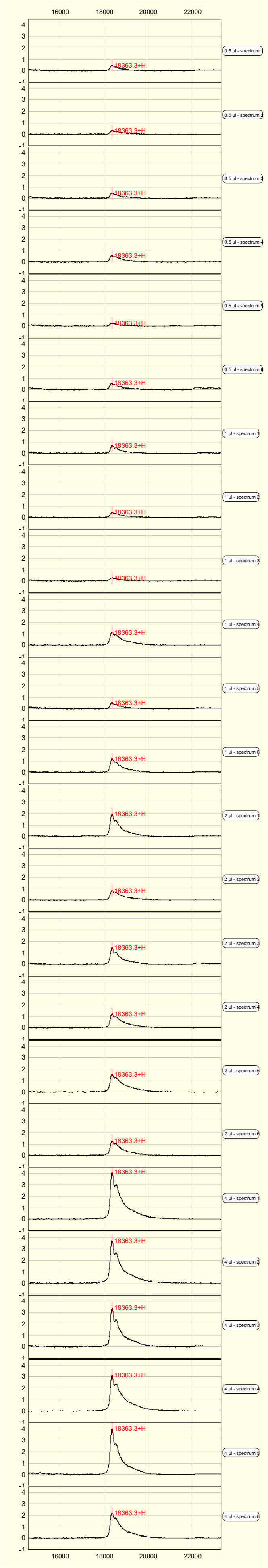

Supplementary Figure 1

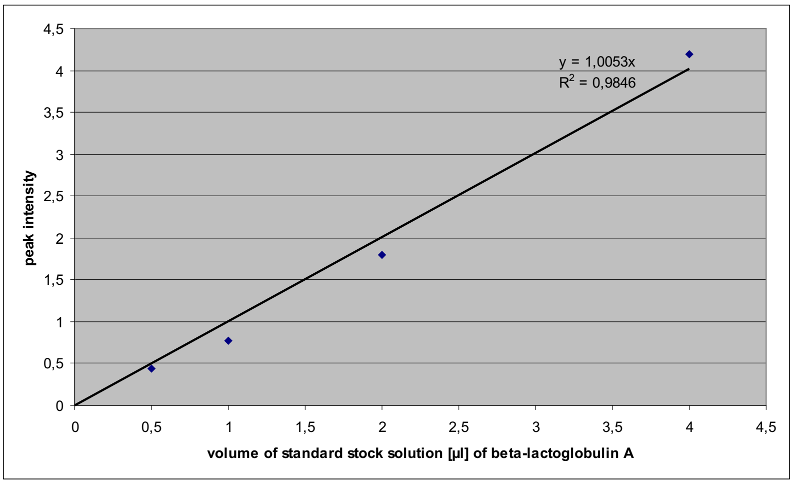

Supplementary Figure 2
