# Supplementary material for: The new platinum-based anticancer agent LA-12 induces retinol binding protein 4 in vivo
Source: Proteome Sci. 2011 Oct 31;9:68. doi: 10.1186/1477-5956-9-68 (PMC3221626; doi:10.1186/1477-5956-9-68)

Additional file 5. Overview of protein fractions obtained by reverse-phase protein fractionation as measured by SELDI-TOF MS (NP-20 surface). Protein with m/z=22684 was detected in fraction No. 11 only and confirmed in a mixture of fractions No. 11,12,13 and 14.

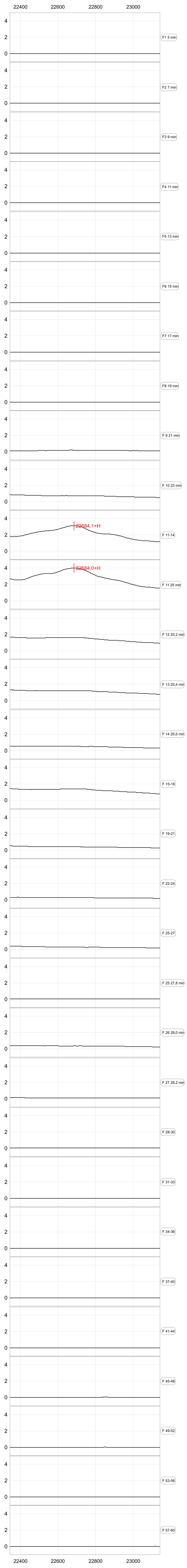

Supplement: Additional file 5 — Overview of protein fractions obtained by reverse-phase protein fractionation as measured by SELDI-TOF MS (NP-20 surface). Overview of protein fractions obtained by reverse-phase protein fractionation as measured by SELDI-TOF MS (NP-20 surface). Protein with m/z = 22684 was detected in fraction 11 only and confirmed in a mixture of fractions 11,12,13 and 14. [file 1477-5956-9-68-S5.PDF]
