# Supplementary material for: The new platinum-based anticancer agent LA-12 induces retinol binding protein 4 in vivo
Source: Proteome Sci. 2011 Oct 31;9:68. doi: 10.1186/1477-5956-9-68 (PMC3221626; doi:10.1186/1477-5956-9-68)
Supplement: Additional file 6 — MS/MS spectra of rat RBP4 identified peptides. Figure A. MS/MS spectrum of peptide m/z = 2079.943. Peptide was selected from MS spectrum of Plasma retinol-binding protein Precursor P04916 (see Figure 3 in the main text). Measured fragment series y and b are marked in spectrum and their m/z values are listed in table together with amino acid sequence of peptide. Asterisk indicates carbamidomethylation of cysteine residue. Figure B. MS/MS spectrum of peptide m/z = 2407.196. Peptide was selected from MS spectrum of Plasma retinol-binding protein precursor P04916 (see Figure 3 in the main text). Measured fragment series y and b are marked in spectrum and their m/z values are listed in table together with amino acid sequence of peptide. Figure C. MS/MS spectrum of peptide m/z = 2705.146. Peptide was selected from MS spectrum of Plasma retinol-binding protein precursor P04916 (see Figure 3 in the main text). Measured fragment series y and b are marked in spectrum and their m/z values are listed in table together with amino acid sequence of peptide. Asterisk indicates carbamidomethylation of cysteine residue. [file 1477-5956-9-68-S6.PDF]

Additional file 6: MS/MS spectra of rat RBP4 identified peptides

Figure A.
MS/MS spectrum of peptide m/z=2079.943. Peptide was selected from MS spectrum of Plasma retinol-binding protein Precursor P04916 (see Fig. 3 in the main text). Measured fragment series *y* and *b* are marked in spectrum and their m/z values are listed in table together with amino acid sequence of peptide. Asterisk indicates carbamidomethylation of cysteine residue.

Figure B.
MS/MS spectrum of peptide m/z=2407.196. Peptide was selected from MS spectrum of Plasma retinol-binding protein precursor P04916 (see Fig. 3 in the main text). Measured fragment series *y* and *b* are marked in spectrum and their m/z values are listed in table together with amino acid sequence of peptide.

Figure C.
MS/MS spectrum of peptide m/z=2705.146. Peptide was selected from MS spectrum of Plasma retinol-binding protein precursor P04916 (see Fig. 3 in the main text). Measured fragment series *y* and *b* are marked in spectrum and their m/z values are listed in table together with amino acid sequence of peptide. Asterisk indicates carbamidomethylation of cysteine residue.

Figure A.

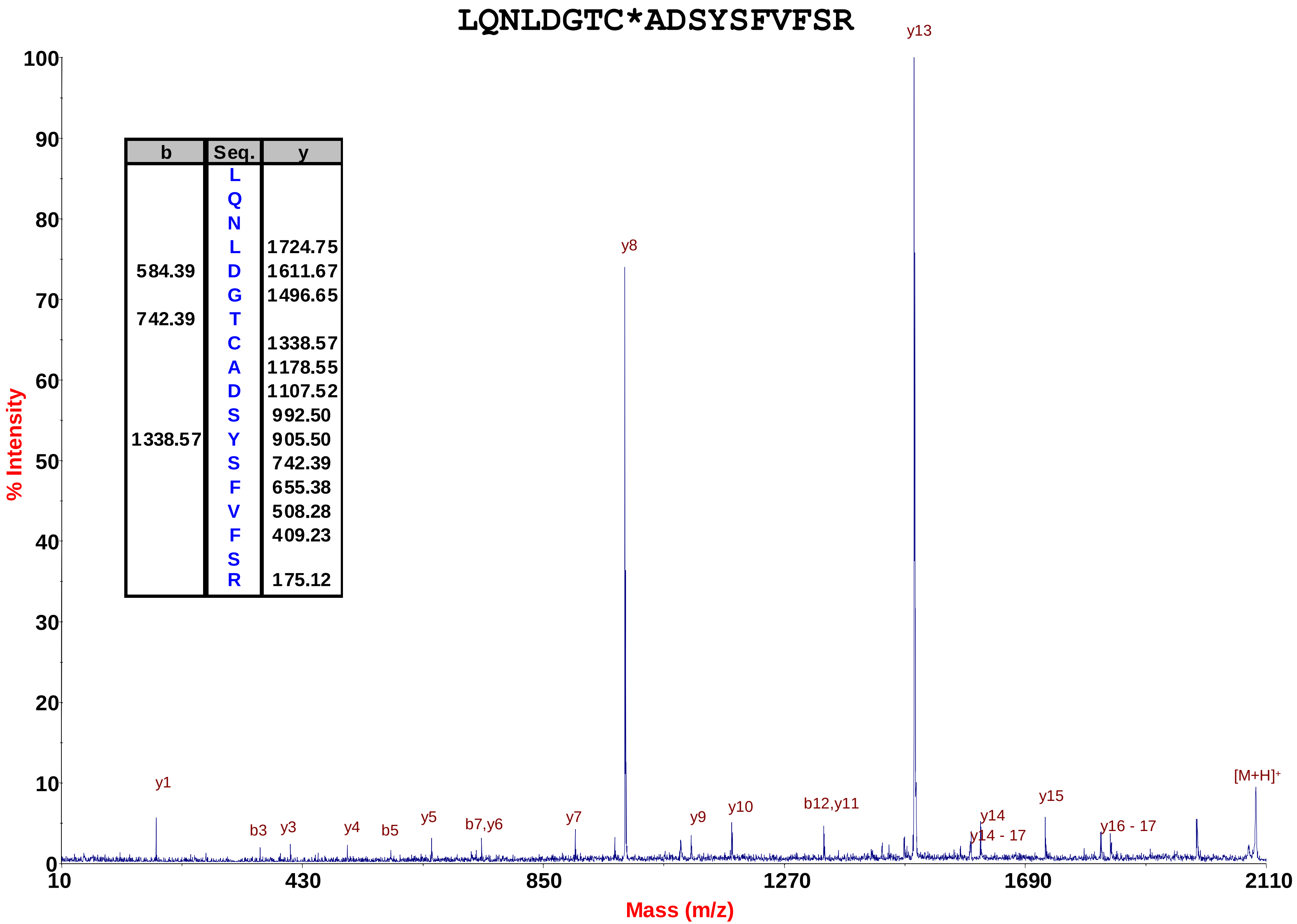

b	Seq.	y
	L	
	Q	
	N	
	L	1724.75
584.39	D	1611.67
	G	1496.65
742.39	T	
	C	1338.57
	A	1178.55
	D	1107.52
	S	992.50
1338.57	Y	905.50
	S	742.39
	F	655.38
	V	508.28
	F	409.23
	S	
	R	175.12

Figure B.

KDPEGLFLQDNIIAEFSVDEK

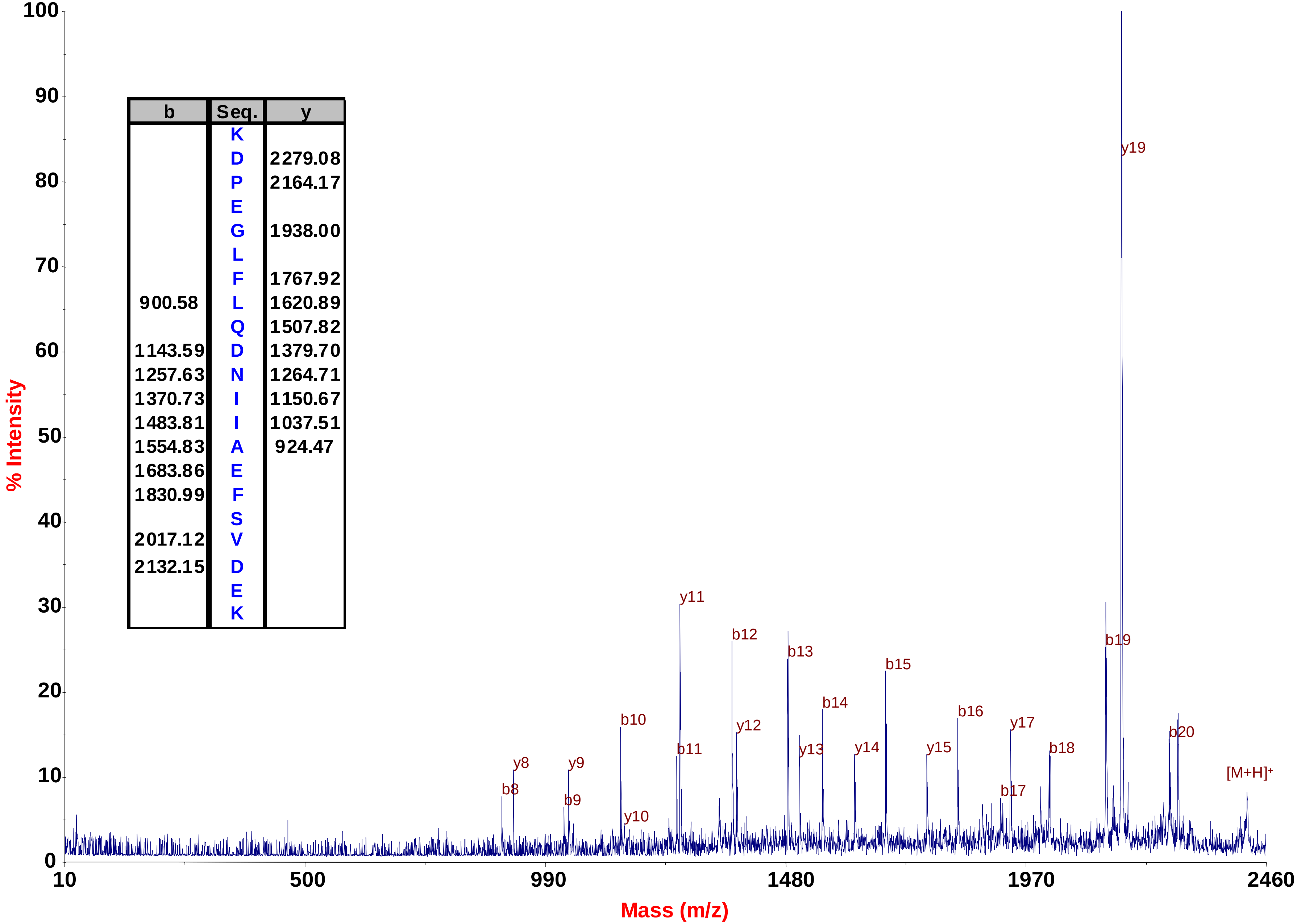

b	Seq.	y
	K	
	D	2279.08
	P	2164.17
	E	
	G	1938.00
	L	
	F	1767.92
900.58	L	1620.89
	Q	1507.82
1143.59	D	1379.70
1257.63	N	1264.71
1370.73	I	1150.67
1483.81	I	1037.51
1554.83	A	924.47
1683.86	E	
1830.99	F	
	S	
2017.12	V	
2132.15	D	
	E	
	K	

Figure C.

GNDDHWIIDTDYDTFALQYSC*R

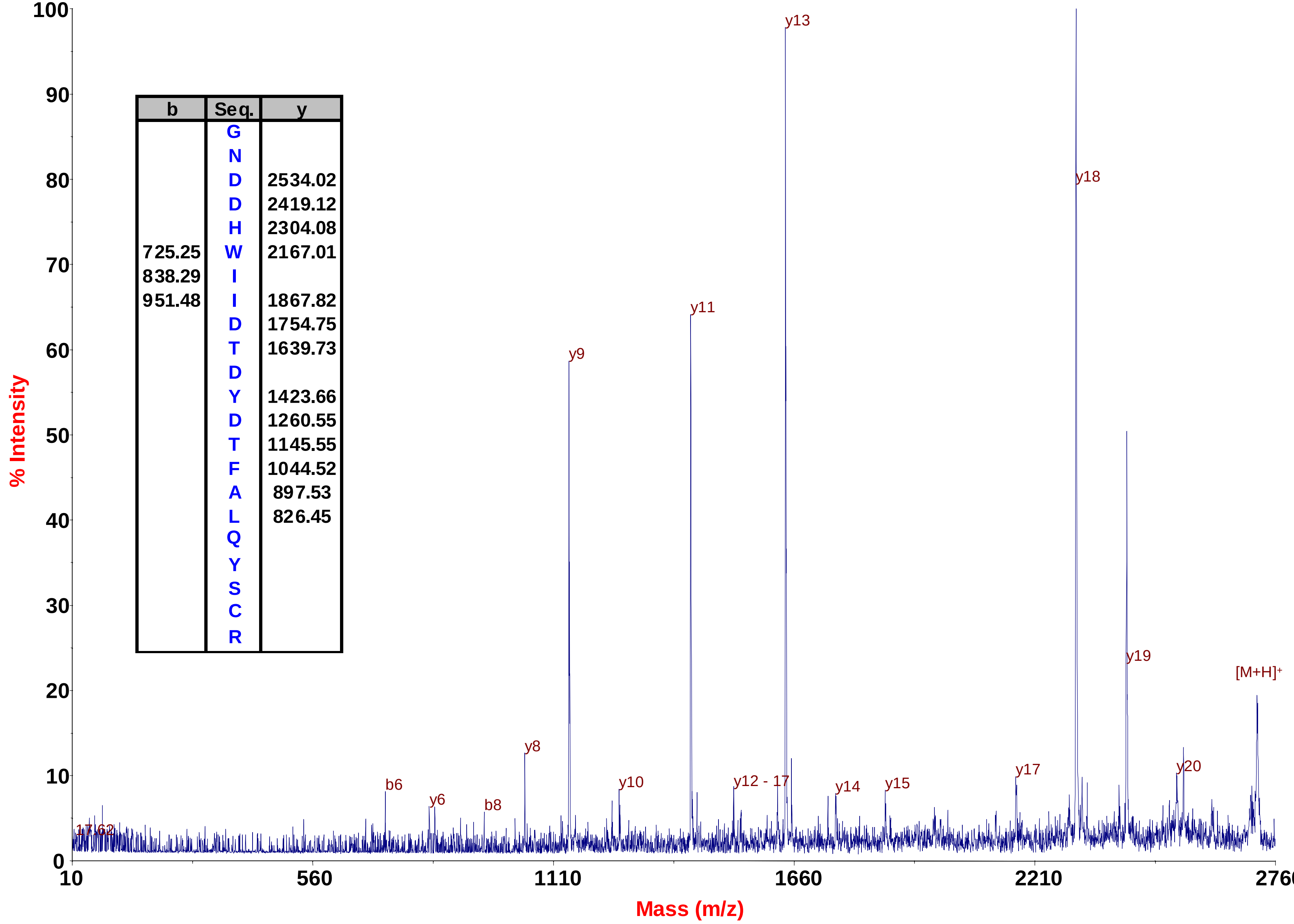

b	Seq.	y
	G	
	N	
	D	2534.02
	D	2419.12
	H	2304.08
725.25	W	2167.01
838.29	I	
951.48	I	1867.82
	D	1754.75
	T	1639.73
	D	
	Y	1423.66
	D	1260.55
	T	1145.55
	F	1044.52
	A	897.53
	L	826.45
	Q	
	Y	
	S	
	C	
	R	
