# Supplementary material for: The new platinum-based anticancer agent LA-12 induces retinol binding protein 4 in vivo
Source: Proteome Sci. 2011 Oct 31;9:68. doi: 10.1186/1477-5956-9-68 (PMC3221626; doi:10.1186/1477-5956-9-68)
Supplement: Additional file 7 — The structural alignment of the ribbon models of the apo structure of human plasma retinol-binding protein (PDB ID 1BRT) and its holo structure (PDB ID 1BRP) in the complex with the native ligand retinol. The apo structure is shown in magenta, the holo structure is in blue and the retinol is colored by atom type. The most significant difference between two forms of the protein is due to the conformational change involving residues from 34 to 37 (black arrow). The figure was prepared using the software PyMol v0.99 (DeLano Scientific LCC, South San Francisco, CA, USA). [file 1477-5956-9-68-S7.PDF]

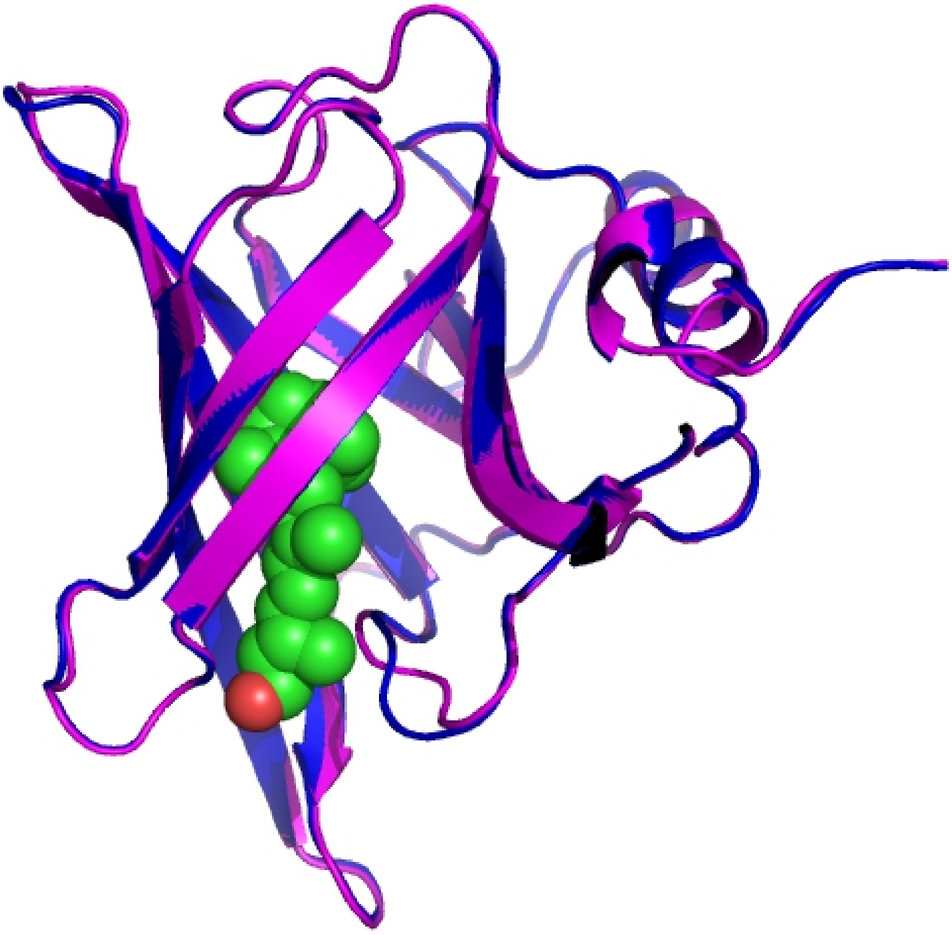

Additional file 7. The structural alignment of the ribbon models of the apo structure of human plasma retinol binding protein (PDB ID 1BRT) and its holo structure (PDB ID 1BRP) in the complex with the native ligand retinol. The apo structure is shown in magenta, the holo structure is in blue and the retinol is colored by atom type. The most significant difference between two forms of the protein is due to the conformational change involving residues from 34 to 37 (black arrow).
The figure was prepared using the software PyMol v0.99 (DeLano Scientific LCC, South San Francisco, CA, USA).
