# Supplementary material for: The new platinum-based anticancer agent LA-12 induces retinol binding protein 4 in vivo
Source: Proteome Sci. 2011 Oct 31;9:68. doi: 10.1186/1477-5956-9-68 (PMC3221626; doi:10.1186/1477-5956-9-68)
Supplement: Additional file 8 — The correlation between platinum and RBP4 protein levels in plasma from 12 patients undergoing the Phase I clinical trials. Each patient is represented by one chart (A-L). Left axis and triangles corresponds with platinum level while right axis and squares stand for RBP4 level. Original western blotting data are presented above each chart. [file 1477-5956-9-68-S8.PDF]

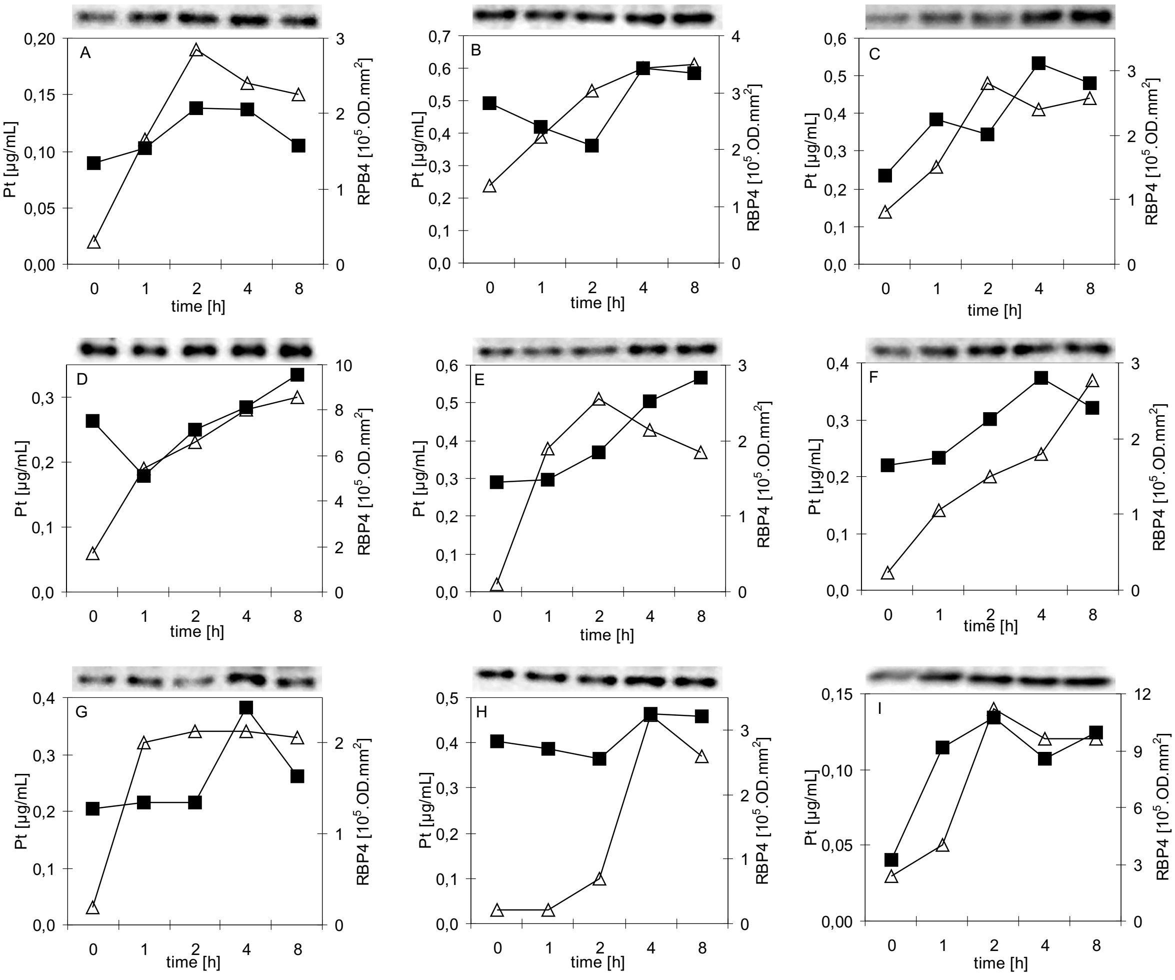

Additional file 8. The correlation between platinum and RBP4 protein levels in plasma from 12 patients undergoing the Phase I of clinical trials. Each patient is represented by one chart (A-L). Left axis and triangles corresponds with platinum level while right axis and squares stand for RBP4 level. Raw western blot data are presented above each chart.
